# Charactering the interaction between expression quantitative trait loci and Alzheimer's Disease Status

**DOI:** 10.1002/alz70855_106835

**Published:** 2025-12-25

**Authors:** Makaela Mews, Yousef Mustafa, Nicholas R. Wheeler, Tianjie Gu, Lissette Gomez, Larry D Adams, Takiyah D. Starks, Mario Cornejo‐Olivas, Maryenela Illanes‐Manrique, Concepcion Silva, Briseida E. Feliciano‐Astacio, Goldie S Byrd, Margaret Pericak‐Vance, Jonathan L Haines, Anthony J Griswold, William S Bush

**Affiliations:** ^1^ Systems Biology & Bioinformatics, Department of Nutrition, School of Medicine, Case Western Reserve University, Cleveland, OH, USA; ^2^ Cleveland Institute for Computational Biology, Department of Population & Quantitative Health Sciences, School of Medicine, Case Western Reserve University, Cleveland, OH, USA; ^3^ John P. Hussman Institute for Human Genomics, University of Miami Miller School of Medicine, Miami, FL, USA; ^4^ Maya Angelou Center for Health Equity, Wake Forest University School of Medicine, Winston‐Salem, NC, USA; ^5^ Neurogenetics Research Center, Instituto Nacional de Ciencias Neurológicas, Lima, Peru; ^6^ Universidad Central del Caribe, Bayamon, PR, USA; ^7^ Univeridad Central del Caribe, Bayamon, PR, USA; ^8^ Wake Forest University School of Medicine, Winston‐Salem, NC, USA; ^9^ Dr. John T. Macdonald Foundation Department of Human Genetics, University of Miami Miller School of Medicine, Miami, FL, USA

## Abstract

**Background:**

While genetic variation is a major determinant of Alzheimer's Disease (AD) risk, changes in gene expression likely associate with the disease process in complex and poorly understood ways. To address this gap, we examined how genetic variants alter whole‐blood gene expression in the context of AD status within African American (AA; *N* = 224), Caribbean Hispanic (CH; *N* = 209), Peruvian (PER; *N* = 83), and Non‐Hispanic White (NHW; *N* = 235) AD cases and controls.

**Method:**

RNAseq data alongside TOPMed‐imputed genotype data was processed using the Alzheimer's Disease Sequencing Project (ADSP) FunGen‐xQTL protocol. We performed expression quantitative trait loci (eQTL) analysis stratified by cohort, including AD status as a base covariate and an interaction term (eQTL*AD), adjusting for sex, age at exam, cohort substructure (PC1‐3), 14 cell‐type proportions calculated (CIBERSORTx), and additional experimental factors.

**Result:**

We identified 68 genes which harbor significant interaction eQTLs (ieQTLs; eQTL*AD), shared amongst at least two cohorts. Notably, we identified CACNG6, which encodes a voltage gated calcium channel, shared among NHW, AA, and PER cohorts. We observed an enrichment for genes regulated by the ZFHX3 and ELF2 transcription factors, which may point to specific immune regulatory mechanisms altered by the Alzheimer's disease process. Our cohort‐specific analyses identified 4,733 significant ieQTLs in AA, 4,322 in NHW, 3,084 in CH, and 1,436 in PER cohorts. All ieQTLs were cohort‐specific at a variant‐level, with 20‐30% overlapping known AD loci.

**Conclusion:**

Our eQTL analysis revealed substantial cohort‐related differences in whole blood gene expression regulation, including numerous cohort‐specific ieQTL effects. These findings suggest that genetic variation may modulate immune‐related pathways relevant to AD pathogenesis. This underscores the need for multi‐cohort studies to inform the development of effective AD therapies.

